# A Treatise on the Diseases of the Mouth, Jaws and Associate Parts

**Published:** 1869-11

**Authors:** 


### BIBLIOGRAPHICAL NOTICE.

*A Treatise on the Diseases of the Mouth, Jaws and Associate Parts.* By James E. Garretson, M. D., D. D. S.,—Publishers, J. B. Lippincott & Co., Philadelphia. This is a volume of seven hundred pages, illustrated by one hundred and twenty wood cuts, and thirteen steel plates, and, as the preface states, embodies the results of the Author's observations and experiences, during a somewhat extended practice in that branch of the profession to which it specially relates.

Chapter I. is devoted to the Surgical Anatomy of the Mouth and Face, and is a clear and well written description of the osseous structure of the skull. The wood cuts illustrating this chapter are well engraved, and we only regret that the style of Gray's Anatomy has not been followed, where instead of numbers, the full name of each part appears upon the part itself without foot notes.

Chapter II. contains a description of the mouth, embracing the muscles, arteries, nerves and veins of the face, sectional expression of the mouth, descriptions of the tongue, mucous membrane, salivary glands and temporo-maxillary articulation.

Chapter III. is devoted to a lengthy and well written description of the Fifth Pair of Nerves, but the arteries supplying the teeth, should, we think, have received the same attention. This, however, is not the case, and from the meager description given in the second chapter, a perfect knowledge of these organs cannot be obtained. The author may, however, reply that this work was not written for an Anatomy, but as he has given us such a complete account of the bones of the face, we were led to expect corresponding ones of the muscles and arteries.

Chapters IV., V. and VI. are upon Dentition, Associate Lesions of First Dentition, and the Anomalies of Second Dentition and their Surgical Relations. These subjects are well defined and illustrated by plates from Forget's description of these anomalies.

Chapter VII. under the general head of the Teeth and their Diseases, treats of Alveolar Abscess, and the Author claims as original the surgical treatment of destroying the sack by drilling through the outer wall of the alveolar cavity with a spear shaped drill, and thus reaching the sack in order to break it up, a method which has been practiced for many years. He very justly, however, denounces the practice of applying blisters and warm fomentations to the face in incipient alveolar abscess, and recommends, where it is not desirable to adopt the surgical method, the application of a roasted fig or raisin directly to the affected part. A number of interesting examples of the anomalies of alveolar abscess, selected from the Author's practice and that of others, are described in this chapter.

Chapter VIII. treats of Trismus. Chapter IX. of Caries, from which a great deal of useful information may be obtained, on the structure and composition of the teeth, and the nature of the food proper in infancy and childhood. This is followed by a brief description of the treatment of caries, with illustrations of the instruments necessary and the materials in use. Also several formulas are recommended for mouth washes and tooth powders.

Chapter X. is devoted to Odontalgia, and the following causes of this affection are named: Sensitive Dentine, Direct or indirect exposure of pulp to sources of irritation, Diseased state of the Periodonteum, Confinement of pus and gas in the pulp cavity, Granules of osteo-dentine in the pulp, Sympathy, Recession and Absorption of the gums and Aveolus.

Chapter XI. describes Salivary Calculus and the method for its removal. Chapter XII. Denudation, the Author asserting that this disease to be one of predisposition, the result of impressions made upon the enamel at the period of its formation, and which predisposition has never by accident or design been corrected. As means of relief from Mechanical Abrasions, he refers to the several methods in use, such as metallic caps on the posterior teeth, and the forming of cavities in the abraded surfaces, which cavities are to be filled with gold so built up as to afford a masticating surface.

Chapters XIII. and XIV. treats of the Extraction of Teeth and the Local Anæsthetics used in this operation, and contains some useful suggestions. Chapters XV. and XVI. are devoted to General Anæsthetics, with a few remarks on Nitrous Oxide Gas, the Author referring the reader to other works for information concerning this agent.

The remaining chapters which compose the greater part of the work, are devoted to Oral Surgery proper, such as Salivary Fistulæ, Affections of the Tonsil Glands, Diseases of the Gums, the significations being accumulation of tartar, periodontitis, the mercurial impressions, scurvy, syphilis, dead or loose teeth, a crowded dental arch, use of improper dentifrices and brushes, improperly inserted artificial teeth, malignant impressions, anomalous conditions; then follow chapters on Caries of the Maxillæ, Necrosis of the Jaws, Tumors of the Mouth, malignant and non-malignant, The Diseases of the Antrum of Highmore, Neuralgia, Wounds of the Mouth and Associate Parts, Ozaena, Fracture of the Maxillary Bones, Dislocation of the Inferior Maxilla, in which Dr. Gunning's splints are described, and brief mention made of Dr. Bean's splint and occipito-frontal bandage, which the Author remarks "is in its character similar in principle to the splint of Dr. Gunning," (an opinion which we think, does not do justice to Dr. Bean), Operations upon the Lips and Cheek, Diseases of the

Tongue, Apthae, Ranula, Palatine Defects and their Treatment, Obturators and Resection of the Maxillary Bones.

College and other duties at this season of the year prevents our giving more than this general review, but we feel satisfied that every dentist will find much that is useful in this work, and that it will be of the greatest service to the dental student. The Publishers deserve credit for the handsome manner in which the work is gotten up, both as regards the text and the engravings with which it is so liberally supplied.

